# Clinical Insights Into the *COL4A3* p.Gly407Arg Variant in Alport Syndrome

**DOI:** 10.34067/KID.0000001184

**Published:** 2026-04-03

**Authors:** Ana Marta Gomes, Cláudia Falcão Reis, Joana Dias, Pedro Moreira Martins, Nuno Medeiros, Graça Ferreira, Vitória Faria, Jorge Malheiro, Carolina Lemos, Daniela Lopes, Idalina Beirão

**Affiliations:** 1 Serviço de Nefrologia, Unidade Local de Saúde Gaia/Espinho, Vila Nova de Gaia, Portugal; 2 Unit for Multidisciplinary Research in Biomedicine (UMIB), School of Medicine and Biomedical Sciences (ICBAS), University of Porto, Porto, Portugal; 3 ITR - Laboratory for Integrative and Translational Research in Population Health, Porto, Portugal; 4 Centro Académico Clínico Egas Moniz Health Alliance, Campus Universitário Santiago, Aveiro, Portugal; 5 Serviço de Genética Médica, Centro de Genética Médica Jacinto Magalhães, Unidade Local de Saúde de Santo António, Porto, Portugal; 6 European Rare Kidney Reference Center (ERKNet), Centro Hospitalar Universitário do Porto, Unidade Local de Saúde de Santo António, Porto, Portugal; 7 Serviço de Oftamologia, Unidade Local de Saúde Gaia/Espinho, Vila Nova de Gaia, Portugal; 8 Serviço de Otorrinolaringologia, Unidade Local de Saúde Gaia/Espinho, Vila Nova de Gaia, Portugal; 9 Serviço de Pediatria, Unidade Local de Saúde Gaia/Espinho, Vila Nova de Gaia, Portugal; 10 Cardiovascular, Renal and Metabolism, Global Medical Affairs, BioPharmaceuticals Medical, AstraZeneca, Gothenburg, Sweden; 11 Instituto de Ciências Biomédicas Abel Salazar, Universidade do Porto, Porto, Portugal; 12 Serviço Nefrologia, Centro Hospitalar Universitário do Porto, Unidade Local de Saúde de Santo António, Porto, Portugal

**Keywords:** Alport syndrome

## Abstract

**Key Points:**

- Phenotypic variability remains significant among patients with the monoallelic *COL4A3* p.Gly407Arg variant, despite shared environmental backgrounds.
- Proteinuria ≥500 mg/g is a strong predictor of renal function decline and adverse outcomes, highlighting its value as a key prognostic marker.
- Early nephrologic monitoring and timely initiation of renoprotective therapies, such as renin-angiotensin-aldosterone system inhibitor may delay progression to kidney failure.

**Background:**

Alport syndrome is a hereditary nephropathy caused by pathogenic variants in the *COL4A3*, *COL4A4*, or *COL4A5* genes, encoding type 4 collagen chains that are essential for glomerular basement membrane integrity. The missense pathogenic variant p.Gly407Arg in *COL4A3* has been reported in Portuguese families, but detailed phenotypic characterization remains limited. This study aimed to describe the renal and extrarenal manifestations and identify predictors of renal outcomes in a cohort of patients carrying the *COL4A3* p.Gly407Arg variant.

**Methods:**

We conducted a retrospective observational study including 62 patients from 25 families followed at a tertiary nephrology center in northern Portugal between 2007 and 2025 with genetically confirmed autosomal dominant Alport syndrome due to monoallelic *COL4A3* p.Gly407Arg pathogenic variant. Demographic, clinical, laboratory, audiologic, and ophthalmologic data were analyzed. Renal events were defined as incident kidney failure, eGFR <15 ml/min per 1.73 m^2^, or eGFR ≥30% decline from baseline sustained over time. Changes in eGFR over time were analyzed using a univariate linear mixed effects model.

**Results:**

At diagnosis, 98% of patients presented with microscopic hematuria, and 53% had proteinuria (urinary protein-creatinine ratio, 240 mg/g; interquartile range, 9–840). The mean eGFR was 94±32 ml/min per 1.73 m^2^. The mean follow-up time in the study (from the initiation of clinical observation until the occurrence of the event or the end of follow-up) was 4.72 years (interquartile range, 2.43–8.43). During a median follow-up of 4.72 years, 16 patients (26%) developed renal events, with 5 (8%) initiating KRT at a mean age of 58 (range, 45–66) years. The mean renal survival was 67 years (63–72). Proteinuria ≥500 mg/g was significantly associated with faster eGFR decline (*P* < 0.001) and a higher risk of renal events (imputed as a time-varying variable, hazard ratio, 1.03; 95% confidence interval, 1.001 to 1.059; *P* = 0.04). Hearing loss occurred in 34% of patients and ocular changes in 18% of the patients evaluated.

**Conclusions:**

Patients carrying monoallelic *COL4A3* p.Gly407Arg pathogenic variant exhibit variable phenotypic expression, with proteinuria representing the strongest predictor of renal function decline.

## Visual Abstract

**Figure:**
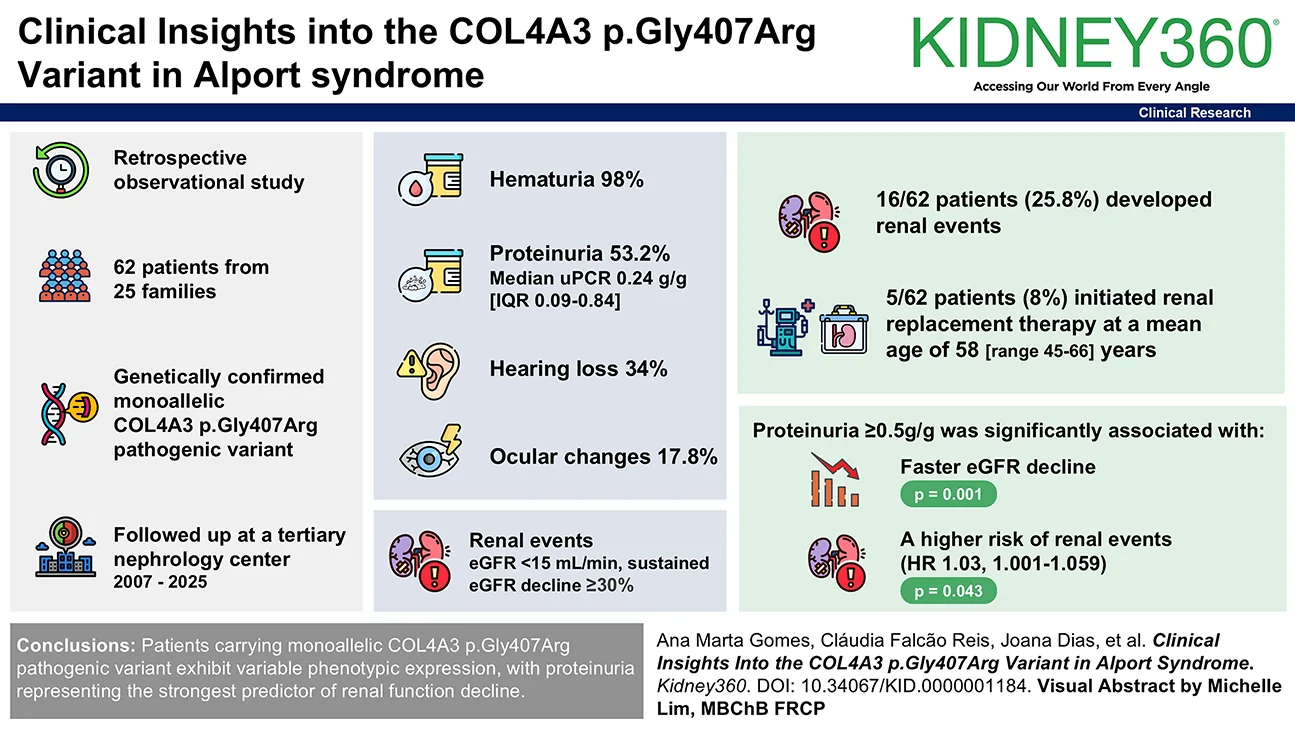

## Introduction

Alport syndrome (AS) is a hereditary kidney disease caused by genetic variants of *COL4A3*, *COL4A4*, and *COL4A5* genes.^1^ These genes encode proteins that form collagen type 4, the major component of the glomerular basement membrane. Collagen type 4 *α*3, *α*4, and *α*5 chains gather in a sequential helical manner from their carboxy-terminal non-collagenous 1 domain, through its collagenous intermediate domains and finally *N*-terminal 7S domains.^2^ The collagenous domain consist of glycine (Gly)-Xaa-Yaa triplet repeats with interruptions that confer flexibility to the molecule.^3^ Gly is the smallest amino acid fitting within the triple helix; thus, its substitution by larger amino acids cause distortion of the protein. Impairment is most severe when Gly is substituted by Arg, Glu, Asp, Val, and Trp^4–6^ causing heterotrimer misfolding and protein retention in the podocyte endoplasmic reticulum. This activates an adaptative response that induces protein degradation and translation inhibition. Prolonged endoplasmic stress becomes cytotoxic, leading to apoptosis.^5^ Clinical manifestations vary according to the mode of genetic inheritance—X-linked, autosomal recessive AS, autosomal dominant AS (ADAS), or digenic,^7^ as well as the location of the variant and its type.^5,8^ In addition, some hypomorphic variants, most commonly in *COL4A5*, result in milder phenotype as male patients required KRT later in life.^4^ The most common hypomorphic variant in *COL4A5* is p.(Gly624Asp) that is particularly found in some European populations.^5,9,10^

Monoallelic deleterious variants of *COL4A3* or *COL4A4*, located at chromosome 2, cause AS due to dominant negative effect, with an autosomal dominant inheritance pattern. Heterozygous *COL4A3* or *COL4A4* variants affect one in 106 patients, and now it is recognized that ADAS is the most common mode of inheritance.^11^ The clinical spectrum of ADAS is varied, ranging from isolated (sometimes intermittent) hematuria^1^ to progressive proteinuria, gradual decline of the GFR, and eventual kidney failure. Asymptomatic individuals heterozygous for pathogenic variants can be observed due to incomplete penetrance.^7^ Extrarenal involvement is uncommon but may include sensorineural deafness and ocular anomalies such as perimacular and peripheral coalescing flecks, temporal retinal thinning, and lenticonus.^12,13^ Moreover, AS exhibits both intrafamilial and interfamilial variable expressivity. Risk factors associated with disease increased severity include smoking, high BP, and high intake of salt and animal proteins.^7^ In our cohort of patients with ADAS, comprising 97 individuals followed at our clinic, 64% (62 patients) carried the pathogenic variant p.Gly407Arg in *COL4A3*. Nabais *et al.* previously reported this variant in eight unrelated Portuguese families from the same region, all of whom were found to segregate identical intragenic *COL4A3* polymorphisms, suggesting a shared common ancestor. A wide spectrum of renal manifestations was observed.^14^

The aim of this study was to characterize in greater detail the renal and extra-renal clinical manifestations in a cohort of patients carrying the *COL4A3* p.Gly407Arg pathogenic variant followed at our institution's Hereditary Nephropathy Outpatient Clinic.

## Methods

### Study Design and Patient Recruitment

A retrospective study was conducted between January 2007 and April 2025 at the Local Health Unit of Vila Nova de Gaia/Espinho, a tertiary care center located in northern Portugal. This institution provides renal care services to an estimated population of approximately 600,000 inhabitants.

This study was conducted in accordance with the principles of the Declaration of Helsinki and was approved by the local ethics committee (Comissão de Ética para a Saúde 152/2021-1). Informed consent was obtained from all participants.

### Study Population

We included 62 patients from 25 families carrying a monoallelic *COL4A3* p.Gly407Arg pathogenic variant who were followed at our Local Health Unit between January 2007 and April 2025. Twenty-five patients were index cases, and the remaining 37 were family members diagnosed following cascade screening of at-risk relatives. Molecular testing was performed using either Sanger sequencing or a next-generation sequencing panel targeting genes, including *COL4A3*, *COL4A4*, and *COL4A5* genes, associated with AS. Clinical data were obtained retrospectively and prospectively through interviews and review of medical records. Collected variables included demographic characteristics, relevant medical history, age at first nephrology evaluation, initiation of renin-angiotensin-aldosterone system inhibitors and sodium-glucose cotransporter 2 inhibitors, laboratory data (GFR,^15^ hematuria, protein-creatinine ratio), age at KRT, and presence of hearing or ocular abnormalities.

Hypertension was defined according to the American College of Cardiology/American Heart Association criteria for adults and by the 95th percentile for age, sex, and height for children. Kidney function was estimated using the CKD Epidemiology Collaboration formula for adults and the Schwartz formula for children. Proteinuria was assessed by the urinary protein-creatinine ratio (UPCR) in a first-morning sample, and hematuria was defined as more than three erythrocytes per high-power field in urinary sediment.

Renal events were defined as the occurrence of any of the following: (*1*) progression to kidney failure, (*2*) a decrease in eGFR to <15 ml/min per 1.73 m^2^, or (*3*) a reduction in eGFR of ≥30% from baseline, considered present when this decline was confirmed in two measurements taken 1 year apart and were sustained over time. Follow-up was calculated from the date of the first nephrology evaluation (study entry) to the occurrence of a renal event or the end of the observation period (April 2025), whichever came first.

Tonal audiometry was performed according to the Bureau International d’Audiophonologie 1997 guidelines to assess hearing thresholds.

Ophthalmologic assessment included a detailed review of the patient's ocular history, visual acuity measurement, intraocular pressure assessment, and slit-lamp biomicroscopy of the anterior segment. Dilated fundus examination was performed in all cases. When indicated, the evaluation was complemented by color fundus photography (Topcon TRC-NW100) and spectral-domain optical coherence tomography (SPECTRALIS, Heidelberg Engineering).

A comprehensive family pedigree was obtained for all families. Genetic counseling was offered to all participants, and genetic testing was performed in a certified laboratory.

### Statistical Analysis

Continuous data were described using mean and SD or median (interquartile range [IQR]), and categorical data were expressed as numbers (and percentages) as appropriate. Categorical data were compared using the Pearson chi-square test or Fisher exact test. Continuous variables with parametric distribution were compared with the Student *t* test or ANOVA test, as appropriate. In the case of continuous variables with asymmetrical distribution, a comparison was performed with the Mann–Whitney *U* test. Overall kidney failure free survival was estimated using Kaplan–Meier curves, and the impact of severe proteinuria on survival, as a time-varying covariate, was explored by Cox proportional hazards model.

Patient eGFR annual change was assessed by univariate linear mixed regression model, that imputed subject-specific random effects (intercept and slope defined as eGFR at baseline and time in years, respectively) on an unstructured covariance matrix. The dependent variable was all eGFR measurements, and each independent variable was entered as two-way interaction terms between them and time (in years).

A two-sided *P* value < 0.05 was considered statistically significant. Analyses were performed using STATA/MP v17 (StataCorp, College Station, TX).

## Results

Our cohort was constituted by 62 patients with molecular diagnosis of ADAS due to the pathogenic variant in heterozygosity p.Gly407Arg in *COLA3*, from 25 families. Thirty-five (56%) were women, the mean age of diagnosis of renal disease was 39±18 years, and the patients were followed until a median age of 50 years (36–61). The mean follow-up time in the study (from the initiation of clinical observation until the occurrence of the event or the end of follow-up) was 4.72 years (IQR, 2.4–8.4). No patients died during follow-up.

### Kidney Disease and Outcomes

At the time of diagnosis of renal disease, 61 patients (98%) had microscopic hematuria, and 33 (53%) patients presented with proteinuria. The median of UPCR was 240 mg/g (9–840), and the mean eGFR was 94±32 ml/min per 1.73 m^2^. Fifty-nine (95%) had a first-degree family history of renal disease. Thirty-nine (63%) patients had arterial hypertension, and four had type 2 diabetes mellitus. Only four patients underwent renal biopsy. In three of these, the biopsy was diagnostic, as electron microscopy revealed basement membrane abnormalities consistent with AS. In one patient with FSGS, the biopsy was nondiagnostic since electronic microscopy was not performed.

Over the course of follow-up, renal events were documented in 16 patients (26%), with five of these patients starting KRT at median age of 58±9 years.

**Figure 1:**
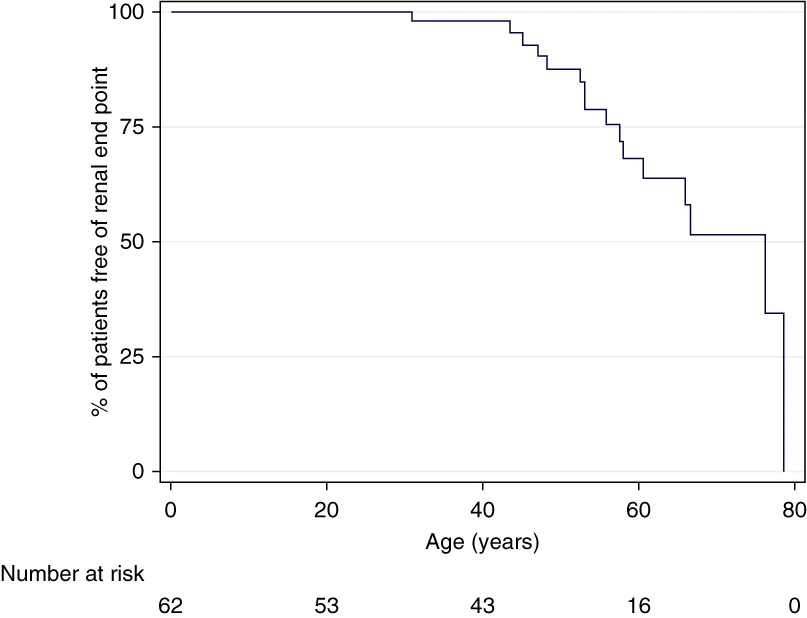
Kaplan–Meier curve of renal survival in patients with monoallelic pathogenic variant in *COL4A3* p.Gly407Arg.

The mean renal survival was 67 years (62–67), as estimated by the Kaplan–Meier method (Figure 1). The mean survival stratified by sex was 64 (57–71) years for men and 70 (64–75) years for women.

In univariate analysis, older age, hypertension, use of nonsteroidal anti-inflammatory drugs, higher level of proteinuria, and lower eGFR rate at diagnosis were identified as the factors most strongly associated with worse renal outcomes (Table 1).

**Table 1:** Baseline characteristics of patients according to renal event status

Characteristic	Total (*N*=62)	Without Renal Event (*n*=46)	With Renal Event (*n*=16)
Age at diagnosis of RD	40±18	37±32	48±13
Female	35 (56%)	29 (63%)	6 (38%)
Family history RD	58 (94%)	45 (98%)	13 (81%)
Hypertension	39 (63%)	24 (52%)	15 (94%)
Median age at diagnosis of RD	46±10	46±11	46±10
Use of RAASi	46 (74%)	32 (70%)	14 (88%)
Average age at initiation of RAASi	45±14	44±14	49±57
Dyslipidemia (*N*=55)	17 (31%)	12 (28%)	5 (42%)
Nonsmoker	47 (76%)	36 (78%)	11 (69%)
Ex-smoker	9 (15%)	5 (11%)	4 (25%)
Smoker	6 (10%)	5 (11%)	1 (6%)
Type 2 diabetes	4 (6%)	3 (7%)	1 (6%)
Renal lithiasis	7 (11%)	4 (9%)	3 (19%)
Use of NSAID	6 (10%)	2 (4%)	4 (25%)
Macroscopic hematuria	5 (8%)	5 (11%)	0 (0%)
UPCR at diagnose RD, mg/g	240 (9–840)	150 (9–360)	1170 (720–1580)
**UPCR at diagnosis of RD, mg\g**			
<150	25 (40%)	23 (50%)	2 (13%)
≥150 to <500	14 (23%)	14 (30%)	0 (0%)
≥500	23 (37%)	9 (20%)	14 (88%)
eGFR at diagnosis RD	94±32	107±21	59±33
eGFR at diagnosis RD<60 ml/min	10 (16%)	2 (4%)	8 (50%)
Follow-up time, yr	4.9 (3.0–11.1)	4.9 (2.7–9.2)	10.1 (3–16.2)

eGFR, eGFR using the CKD Epidemiology Collaboration equation; NSAID, nonsteroidal anti-inflammatory drug; RAASi, renin-angiotensin-aldosterone system inhibitor; RD, renal disease; UPCR, urinary protein-creatinine ratio.

The progression of proteinuria over time showed a gradual increase in its median value, as presented in Table 2. At the end of follow-up, among patients without a renal event, 41 (66%) were receiving renin-angiotensin-aldosterone system inhibitor, with a median age at initiation of 44 years (IQR, 38–54). In this group, 21 patients (34%) were treated with sodium-glucose cotransporter 2 inhibitors, prescribed at a median age of 55 years (IQR, 50–59) to improve proteinuria control.

**Table 2:** Progression of proteinuria over 10 years

Year of Follow-Up	Median UPCR (IQR)	No. of Patients
1	190 (111–890)	62
2	300 (130–1140)	41
3	310 (100–620)	38
4	360 (100–720)	30
5	160 (9–510)	26
6	650 (230–1670)	18
7	740 (370–1820)	18
8	630 (290–1140)	12
9	800 (260–1230)	12
10	1470 (21–1960)	8

IQR, interquartile range; UPCR, urinary protein-creatinine ratio.

**Figure 2:**
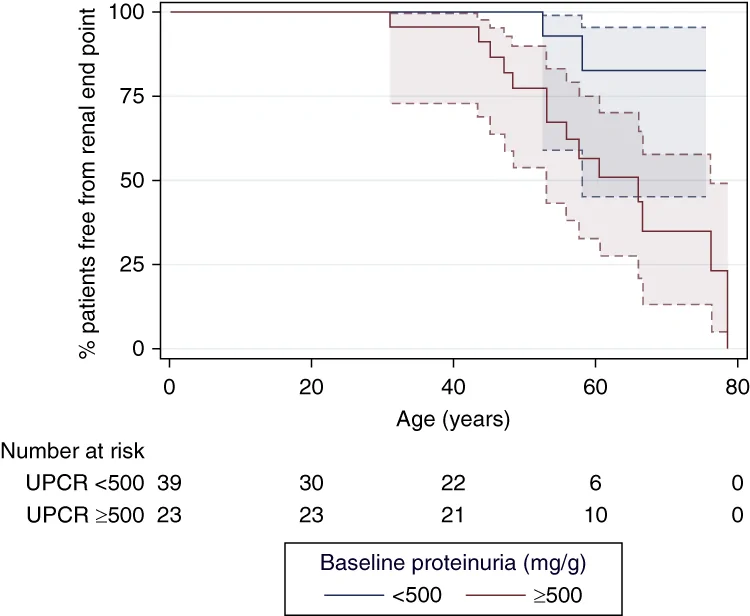
**Kaplan–Meier–type survival curve for the renal end point according to proteinuria status (UPCR <500 versus ≥500 mg/g), modeled as a time-varying covariate in a Cox proportional hazards analysis.** UPCR, urinary protein-creatinine ratio.

In a Cox proportional hazards model incorporating proteinuria status (UPCR <500 versus ≥500 mg/g) as a time-varying covariate, higher proteinuria was significantly associated with an increased risk of reaching the renal end point (hazard ratio, 1.03; 95% confidence interval [CI], 1.00 to 1.06; *P* = 0.04; Figure 2). The mean eGFR decline over time, analyzed using a univariate linear mixed-effects model, was −1.74 ml/min per 1.73 m^2^ per year (95% CI, −2.16 to −1.31; Table 3). The decline was more pronounced in patients with UPCR ≥500 mg/g (−2.71; 95% CI, −3.18 to −2.23) and showed a similar trend in patients with baseline eGFR <60 ml/min per 1.73 m^2^ (−2.47; 95% CI, −3.64 to −1.29). No significant association was observed between the slope of eGFR decline and extra-renal^12^ features.

**Table 3:** Changes in eGFR over time analyzed using a univariate linear mixed-effects model

Characteristic	Slope (95% CI), ml/min per Year	Diff (95% CI)	*P* Value
Overall	−1.74 (−2.16 to −1.31)	NA	<0.001
Age <40	−1.45 (−1.97 to 0.92)	−0.44 (−1.27 to 0.38)	0.29
Age ≥40	−1.89 (−2.52 to −1.26)		
Female	−1.79 (−2.45 to 1.13)	+0.11 (−0.77 to 0.98)	0.81
Male	−1.68 (−2.26 to −1.10)		
Without hypertension	−1.64 (−2.86 to −0.41)	−0.26 (−1.57 to 1.05)	0.70
With hypertension	−1.90 (−2.35 to −1.44)		
Without RAAi	−0.80 (−2.37 to 0.76)	−1.07 (−2.70 to 0.56)	0.20
With RAAi	−1.88 (−2.31 to −1.44)		
**UPCR at diagnose, mg/g**			
<150	−1.16 (−1.9 to −0.38)	Ref	
≥150 to <500	−1.21 (−2.33 to −0.09)	−0.05 (−1.41 to 1.32)	0.95
≥500	−2.71 (−3.18 to −2.23)	−1.54 (−2.4 to 0.63)	<0.001
eGFR at diagnose ≥60 ml/min	−1.59 (−1.99 to −1.19)	−0.88 (−2.12 to 0.36)	0.17
eGFR at diagnose <60 ml/min	−2.47 (−3.64 to −1.29)		
**SNHL (*n*=38)**			
Without	−2.11 (−2.8 to −1.42)	+0.10 (−1.07 to 1.27)	0.87
With	−2.01 (−2.95 to −1.08)		
**Ocular features (*n*=45)**			
Without	−1.58 (−2.15 to −1.00)	−0.78 (−1.83 to 0.27)	0.15
With	−2.35 (−3.23 to −1.47)		

Values represent the estimated effect of time on eGFR. CI, confidence interval; eGFR, eGFR using the CKD Epidemiology Collaboration equation; NA, not applicable; RAAi, renin-angiotensin-aldosterone inhibitor; SNHL, sensorineural hearing loss; UPCR, urinary protein-creatinine ratio.

### Extra-Renal Manifestations

Audiometry evaluation was performed in 38 patients, of whom 13 patients (34%) had bilateral hearing loss at median age of 63 years (59–70). Among these, eight patients had mild bilateral hearing loss, median age 66 years (60–71) and five patients had moderate hearing loss at a median of 60 years (59–64).

Ophthalmic assessment was concluded in 45 patients, and eight patients (18%) had retinal changes associated with AS. No corneal or crystalline lens abnormalities related to AS were observed.

## Discussion

This study provides a comprehensive description of the clinical and renal outcomes of 62 individuals from 25 families carrying the same heterozygous pathogenic variant in *COL4A3* p.Gly407Arg, all originating from the same geographic region. This unique design, combining genetic homogeneity with longitudinal follow-up, offers valuable insights into the phenotypic spectrum and natural history of ADAS related to this variant. Despite sharing an identical pathogenic variant, we observed substantial clinical heterogeneity, consistent with the broad phenotypic variability previously reported in ADAS cohorts.

The natural history of ADAS is characterized by persistent microscopic hematuria, the later development of proteinuria, and a gradual decline in renal function, which may eventually lead to kidney failure in late adulthood. In our cohort, all but one patient exhibited microscopic hematuria at diagnosis, and about half had proteinuria, which aligns with previously reported frequencies ranging from 17% to 72% depending on the age and diagnostic threshold.^12,13,16–19^ Proteinuria emerged as the most important prognostic factor for disease progression. Higher proteinuria levels (≥500 mg/g) were strongly associated with a faster eGFR decline and increased risk of adverse renal outcomes. This observation mirrors previous reports highlighting proteinuria as the primary predictor of renal survival in ADAS.^13,20^

The mean annual decline in eGFR (−1.74 ml/min per 1.73 m^2^ per year) falls within the range described for heterozygous collagen IV nephropathies, typically between −1.0 and −2.5 ml/min per 1.73 m^2^ per year.^12,13,19^ Collectively, these findings support the notion that although ADAS generally progresses slowly, significant renal function loss can occur, especially in patients with sustained proteinuria or hypertension. The mean estimated kidney survival of 67 years observed here is comparable with other cohorts, in which kidney survival typically ranges between 65 and 70 years.^13,18^ Similarly, the overall incidence of renal events (26%) and requirement for KRT (8%) were consistent with previously published studies.^13,17–19^

Sensorineural hearing loss was documented in approximately one-third of evaluated patients, and retinal changes in about one-fifth, while no corneal or lenticular abnormalities were observed. These frequencies are slightly higher than those described in previous ADAS cohorts, where hearing loss is reported in 8%–25% and ocular changes in <5%. This difference may reflect older age at evaluation, referral bias, or enhanced detection methods. As in previous studies, extra-renal manifestations did not correlate with the rate of eGFR decline.^17,19^

Our cohort differs from most published series in several respects. First, all participants share the same *COL4A3* missense variant, providing a genetically homogeneous model to examine phenotypic variability. Second, all families originate from the same geographical region, reducing environmental variability but introducing potential selection bias, since families with known kidney disease are more likely to undergo genetic testing. Indeed, 95% of participants reported a first-degree family history of renal disease, suggesting an overrepresentation of clinically manifest cases. Third, the mean follow-up time of 4.72 years, while informative, captures only part of the disease trajectory, given the typically slow progression of ADAS. As such, our results may underestimate the eventual incidence of kidney failure. Despite these limitations, the uniform genetic background and shared environmental context strengthen the internal consistency of our observations. The coexistence of mild and severe phenotypes within the same families further supports the contribution of additional genetic or environmental modifiers to disease expression—a hypothesis widely acknowledged in recent literature.^5,13,17,19^

Our findings underscore the need for lifelong nephrologic surveillance of *COL4A3* heterozygotes, even in those presenting solely with microscopic hematuria. Proteinuria, when present, should prompt early initiation of renoprotective therapy.

This study has several limitations. Its retrospective design and relatively short follow-up may underestimate long-term outcomes. The inclusion of patients from a single geographic region introduces potential selection bias, and the small number of renal biopsies limits histopathologic correlation. Moreover, therapeutic interventions were not standardized, and potential confounding by indication cannot be excluded. Future research should focus on prospective, multicentric longitudinal cohorts incorporating standardized treatment protocols, exploration of modifier genes, and functional studies to clarify the molecular mechanisms underlying phenotypic heterogeneity in ADAS.

In summary, this study characterizes the clinical course of a genetically homogeneous cohort of patients carrying the *COL4A3* p.Gly407Arg variant, confirming that ADAS is associated with variable expressivity despite identical genotypes. Persistent hematuria and progressive proteinuria remain hallmark features, and proteinuria is the strongest predictor of renal outcome. Although renal survival is generally prolonged, a subset of patients progresses to kidney failure in late adulthood. These findings reinforce the importance of early detection, close monitoring, and timely initiation of renoprotective therapy in all *COL4A3* variant carriers. Continued long-term follow-up and the identification of genetic or environmental modifiers are essential to refine risk stratification and improve personalized care for patients with ADAS.

## Supplementary Material

## Data Availability

Original data generated for the study will be made available upon reasonable request to the corresponding author. Data Type: Observational Data. Reason for Restricted Access: The article contains data that cannot be made publicly available due to confidentiality and ethical restrictions.

## Declarative Statements

This study received Institutional Review Board or Ethics Committee approval. All patients provided written informed consent. This study includes clinical experimentation and complies with the Declaration of Helsinki.
