# Supplementary material for: Clinical Insights Into the COL4A3 p.Gly407Arg Variant in Alport Syndrome
Source: Kidney360. 2026 Apr 3;7(8):1835–41. doi: 10.34067/KID.0000001184 (PMC13567878; doi:10.34067/KID.0000001184)
Supplement: Supplementary file 1 [file kidney360-7-1835-s001.pdf]

ASN American Society of Nephrology
JASN Journal of the American Society of Nephrology
KIDNEY360 Accessing Our World From Every Angle
CJASN Clinical Journal of the American Society of Nephrology

ASN Journal Disclosure Form

As per ASN journal policy, I have disclosed any financial relationships or commitments I have held in the past 36 months as included below. I have listed my Current Employer below to indicate there is a relationship requiring disclosure. If no relationship exists, my Current Employer is not listed.

I. Beirão reports the following:
Employer: Centro hospitalar do Porto

I understand that the information above will be published within the journal article, if accepted, and that failure to comply and/or to accurately and completely report the potential financial conflicts of interest could lead to the following: 1) Prior to publication, article rejection, or 2) Post-publication, sanctions ranging from, but not limited to, issuing a correction, reporting the inaccurate information to the authors' institution, banning authors from submitting work to ASN journals for varying lengths of time, and/or retraction of the published work.

Name: Idalina Beirão
Manuscript ID: K360-2025-001420R1
Manuscript Title: Clinical Insights into the COL4A3 p.Gly407Arg Variant in Alport syndrome.
Date of Completion: February 19, 2026
Disclosure Updated Date: February 10, 2026

ASN American Society of Nephrology | JASN Journal of the American Society of Nephrology | KIDNEY360 Accessing Our World From Every Angle | CJASN Clinical Journal of the American Society of Nephrology

ASN Journal Disclosure Form

As per ASN journal policy, I have disclosed any financial relationships or commitments I have held in the past 36 months as included below. I have listed my Current Employer below to indicate there is a relationship requiring disclosure. If no relationship exists, my Current Employer is not listed.

J. Dias reports the following:
Employer: Unidade Local de Saúde Vila Nova de Gaia/Espinho

I understand that the information above will be published within the journal article, if accepted, and that failure to comply and/or to accurately and completely report the potential financial conflicts of interest could lead to the following: 1) Prior to publication, article rejection, or 2) Post-publication, sanctions ranging from, but not limited to, issuing a correction, reporting the inaccurate information to the authors' institution, banning authors from submitting work to ASN journals for varying lengths of time, and/or retraction of the published work.

Name: Joana Pereira Dias
Manuscript ID: K360-2025-001420R1
Manuscript Title: Clinical Insights into the COL4A3 p.Gly407Arg Variant in Alport syndrome.
Date of Completion: February 10, 2026
Disclosure Updated Date: February 10, 2026

ASN American Society of Nephrology | JASN Journal of the American Society of Nephrology | KIDNEY360 Accessing Our World From Every Angle | CJASN Clinical Journal of the American Society of Nephrology

ASN Journal Disclosure Form

As per ASN journal policy, I have disclosed any financial relationships or commitments I have held in the past 36 months as included below. I have listed my Current Employer below to indicate there is a relationship requiring disclosure. If no relationship exists, my Current Employer is not listed.

G. Ferreira has nothing to disclose.

I understand that the information above will be published within the journal article, if accepted, and that failure to comply and/or to accurately and completely report the potential financial conflicts of interest could lead to the following: 1) Prior to publication, article rejection, or 2) Post-publication, sanctions ranging from, but not limited to, issuing a correction, reporting the inaccurate information to the authors' institution, banning authors from submitting work to ASN journals for varying lengths of time, and/or retraction of the published work.

Name: Graça Ferreira
Manuscript ID: K360-2025-001420R1
Manuscript Title: Clinical Insights into the COL4A3 p.Gly407Arg Variant in Alport syndrome,
Date of Completion: February 19, 2026
Disclosure Updated Date: February 19, 2026

ASN American Society of Nephrology JASN Journal of the American Society of Nephrology KIDNEY360 Accessing Our World From Every Angle CJASN Clinical Journal of the American Society of Nephrology

ASN Journal Disclosure Form

As per ASN journal policy, I have disclosed any financial relationships or commitments I have held in the past 36 months as included below. I have listed my Current Employer below to indicate there is a relationship requiring disclosure. If no relationship exists, my Current Employer is not listed.

A. Gomes reports the following:
Advisory or Leadership Role: CSL Vifor, Novartis; and Speakers Bureau: CSL Vifor.

I understand that the information above will be published within the journal article, if accepted, and that failure to comply and/or to accurately and completely report the potential financial conflicts of interest could lead to the following: 1) Prior to publication, article rejection, or 2) Post-publication, sanctions ranging from, but not limited to, issuing a correction, reporting the inaccurate information to the authors' institution, banning authors from submitting work to ASN journals for varying lengths of time, and/or retraction of the published work.

Name: Ana Marta Gomes
Manuscript ID: K360-2025-001420R1
Manuscript Title: Clinical Insights into the COL4A3 p.Gly407Arg Variant in Alport syndrome
Date of Completion: February 10, 2026
Disclosure Updated Date: February 10, 2026

ASN Journal Disclosure Form

As per ASN journal policy, I have disclosed any financial relationships or commitments I have held in the past 36 months as included below. I have listed my Current Employer below to indicate there is a relationship requiring disclosure. If no relationship exists, my Current Employer is not listed.

C. Lemos reports the following:
Research Funding: ABBVIE; NOVARTIS

I understand that the information above will be published within the journal article, if accepted, and that failure to comply and/or to accurately and completely report the potential financial conflicts of interest could lead to the following: 1) Prior to publication, article rejection, or 2) Post-publication, sanctions ranging from, but not limited to, issuing a correction, reporting the inaccurate information to the authors' institution, banning authors from submitting work to ASN journals for varying lengths of time, and/or retraction of the published work.

Name: Carolina Lemos
Manuscript ID: K360-2025-001420R1
Manuscript Title: Clinical Insights into the COL4A3 p.Gly407Arg Variant in Alport syndrome
Date of Completion: February 11, 2026
Disclosure Updated Date: February 11, 2026

ASN American Society of Nephrology | JASN Journal of the American Society of Nephrology | KIDNEY360 Accessing Our World From Every Angle | CJASN Clinical Journal of the American Society of Nephrology

ASN Journal Disclosure Form

As per ASN journal policy, I have disclosed any financial relationships or commitments I have held in the past 36 months as included below. I have listed my Current Employer below to indicate there is a relationship requiring disclosure. If no relationship exists, my Current Employer is not listed.

D. Lopes has nothing to disclose.

I understand that the information above will be published within the journal article, if accepted, and that failure to comply and/or to accurately and completely report the potential financial conflicts of interest could lead to the following: 1) Prior to publication, article rejection, or 2) Post-publication, sanctions ranging from, but not limited to, issuing a correction, reporting the inaccurate information to the authors' institution, banning authors from submitting work to ASN journals for varying lengths of time, and/or retraction of the published work.

Name: Daniela Machado Lopes
Manuscript ID: K360-2025-001420R1
Manuscript Title: Clinical Insights into the COL4A3 p.Gly407Arg Variant in Alport syndrome
Date of Completion: February 11, 2026
Disclosure Updated Date: February 11, 2026

ASN American Society of Nephrology
JASN Journal of the American Society of Nephrology
KIDNEY360 Accessing Our World From Every Angle
CJASN Clinical Journal of the American Society of Nephrology

ASN Journal Disclosure Form

As per ASN journal policy, I have disclosed any financial relationships or commitments I have held in the past 36 months as included below. I have listed my Current Employer below to indicate there is a relationship requiring disclosure. If no relationship exists, my Current Employer is not listed.

J. Malheiro reports the following:
Employer: AstraZeneca Global; Consultancy: AstraZeneca Global; Honoraria: AstraZeneca Global; Speakers Bureau: AstraZeneca Global; and Other Interests or Relationships: AstraZeneca Global.

I understand that the information above will be published within the journal article, if accepted, and that failure to comply and/or to accurately and completely report the potential financial conflicts of interest could lead to the following: 1) Prior to publication, article rejection, or 2) Post-publication, sanctions ranging from, but not limited to, issuing a correction, reporting the inaccurate information to the authors' institution, banning authors from submitting work to ASN journals for varying lengths of time, and/or retraction of the published work.

Name: Jorge Malheiro
Manuscript ID: K360-2025-001420R1
Manuscript Title: Clinical Insights into the COL4A3 p.Gly407Arg Variant in Alport syndrome
Date of Completion: February 11, 2026
Disclosure Updated Date: February 11, 2026

ASN Journal Disclosure Form

As per ASN journal policy, I have disclosed any financial relationships or commitments I have held in the past 36 months as included below. I have listed my Current Employer below to indicate there is a relationship requiring disclosure. If no relationship exists, my Current Employer is not listed.

P. Martins reports the following:
Employer: ULSGE

I understand that the information above will be published within the journal article, if accepted, and that failure to comply and/or to accurately and completely report the potential financial conflicts of interest could lead to the following: 1) Prior to publication, article rejection, or 2) Post-publication, sanctions ranging from, but not limited to, issuing a correction, reporting the inaccurate information to the authors' institution, banning authors from submitting work to ASN journals for varying lengths of time, and/or retraction of the published work.

Name: Pedro Martins
Manuscript ID: K360-2025-001420R1
Manuscript Title: Clinical Insights into the COL4A3 p.Gly407Arg Variant in Alport syndrome
Date of Completion: February 11, 2026
Disclosure Updated Date: February 11, 2026

ASN American Society of Nephrology
JASN Journal of the American Society of Nephrology
KIDNEY360 Accessing Our World From Every Angle
CJASN Clinical Journal of the American Society of Nephrology

ASN Journal Disclosure Form

As per ASN journal policy, I have disclosed any financial relationships or commitments I have held in the past 36 months as included below. I have listed my Current Employer below to indicate there is a relationship requiring disclosure. If no relationship exists, my Current Employer is not listed.

N. Medeiros reports the following:
Employer: ULSGE

I understand that the information above will be published within the journal article, if accepted, and that failure to comply and/or to accurately and completely report the potential financial conflicts of interest could lead to the following: 1) Prior to publication, article rejection, or 2) Post-publication, sanctions ranging from, but not limited to, issuing a correction, reporting the inaccurate information to the authors' institution, banning authors from submitting work to ASN journals for varying lengths of time, and/or retraction of the published work.

Name: Nuno Medeiros
Manuscript ID: K360-2025-001420R1
Manuscript Title: Clinical Insights into the COL4A3 p.Gly407Arg Variant in Alport syndrome
Date of Completion: February 11, 2026
Disclosure Updated Date: February 11, 2026

ASN Journal Disclosure Form

As per ASN journal policy, I have disclosed any financial relationships or commitments I have held in the past 36 months as included below. I have listed my Current Employer below to indicate there is a relationship requiring disclosure. If no relationship exists, my Current Employer is not listed.

V. Paes de Faria reports the following:
Employer: Unidade Local de Saúde Gaia/Espinho; and Speakers Bureau: Astrazenca.

I understand that the information above will be published within the journal article, if accepted, and that failure to comply and/or to accurately and completely report the potential financial conflicts of interest could lead to the following: 1) Prior to publication, article rejection, or 2) Post-publication, sanctions ranging from, but not limited to, issuing a correction, reporting the inaccurate information to the authors' institution, banning authors from submitting work to ASN journals for varying lengths of time, and/or retraction of the published work.

Name: Vitória Vaz da Rocha Paes de Faria
Manuscript ID: K360-2025-001420R1
Manuscript Title: Clinical Insights into the COL4A3 p.Gly407Arg Variant in Alport syndrome
Date of Completion: February 10, 2026
Disclosure Updated Date: February 10, 2026

ASN
American Society of Nephrology
JASN
Journal of the American Society of Nephrology
KIDNEY360
Accessing Our World From Every Angle
CJASN
Clinical Journal of the American Society of Nephrology

ASN Journal Disclosure Form

As per ASN journal policy, I have disclosed any financial relationships or commitments I have held in the past 36 months as included below. I have listed my Current Employer below to indicate there is a relationship requiring disclosure. If no relationship exists, my Current Employer is not listed.

C. Reis reports the following:
Employer: Hospital de Santo Espírito da Ilha Terceira

I understand that the information above will be published within the journal article, if accepted, and that failure to comply and/or to accurately and completely report the potential financial conflicts of interest could lead to the following: 1) Prior to publication, article rejection, or 2) Post-publication, sanctions ranging from, but not limited to, issuing a correction, reporting the inaccurate information to the authors' institution, banning authors from submitting work to ASN journals for varying lengths of time, and/or retraction of the published work.

Name: Claudai Falcão Reis
Manuscript ID: K360-2025-001420R1
Manuscript Title: Clinical Insights into the COL4A3 p.Gly407Arg Variant in Alport syndrome
Date of Completion: February 18, 2026
Disclosure Updated Date: February 15, 2026
